# A case of lung tumorlets secondary to pulmonary hypoplasia with recurrent haemoptysis

**DOI:** 10.1002/rcr2.373

**Published:** 2018-09-28

**Authors:** Kyoko Yagyu, Atsushi Miyamoto, Haruhiko Matsushita, Akira Okimora

**Affiliations:** ^1^ Department of Respiratory Medicine Izumi City General Hospital Japan; ^2^ Department of Diagnostic Pathology Izumi City General Hospital Japan

**Keywords:** Haemoptysis, pulmonary hypoplasia, tumorlet

## Abstract

Most patients with lung tumorlets are usually asymptomatic, and most diagnoses are incidental findings during microscopic lung examinations at autopsy or after excision of a tissue lesion. A 73‐year‐old woman was admitted to our hospital due to recurrent haemoptysis. Chest computed tomography demonstrated right inferior lobe collapse with bronchiectasis. Bronchoscopic examination revealed the right inferior lobar bronchus to be filled with blood clots. Haemoptysis persisted even after two arterial embolization trials and occlusion with endobronchial Watanabe spigot. Therefore, right lower lobectomy was performed, and multiple tumorlets on lobar hypoplasia were observed on histopathological examination of the excised specimen. We believe that the haemoptysis in our patient was possibly caused by the lung tumorlets secondary to lobar hypoplasia.

## Introduction

Patients with pulmonary tumorlets are usually asymptomatic or present with bronchiectasis symptoms such as cough or recurrent pulmonary infection. Tumorlets are associated with a generally good prognosis 1. Pulmonary hypoplasia is rare in adulthood; a majority of patients present with severe respiratory dysfunction or recurrent infections and commonly have short life expectancies, although some may be asymptomatic and live long 2.

We recently encountered a patient with pulmonary tumorlet with pulmonary hypoplasia requiring emergency surgery due to recurring haemoptysis progressing over a course of five years.

## Case Report

**Figure 1:**
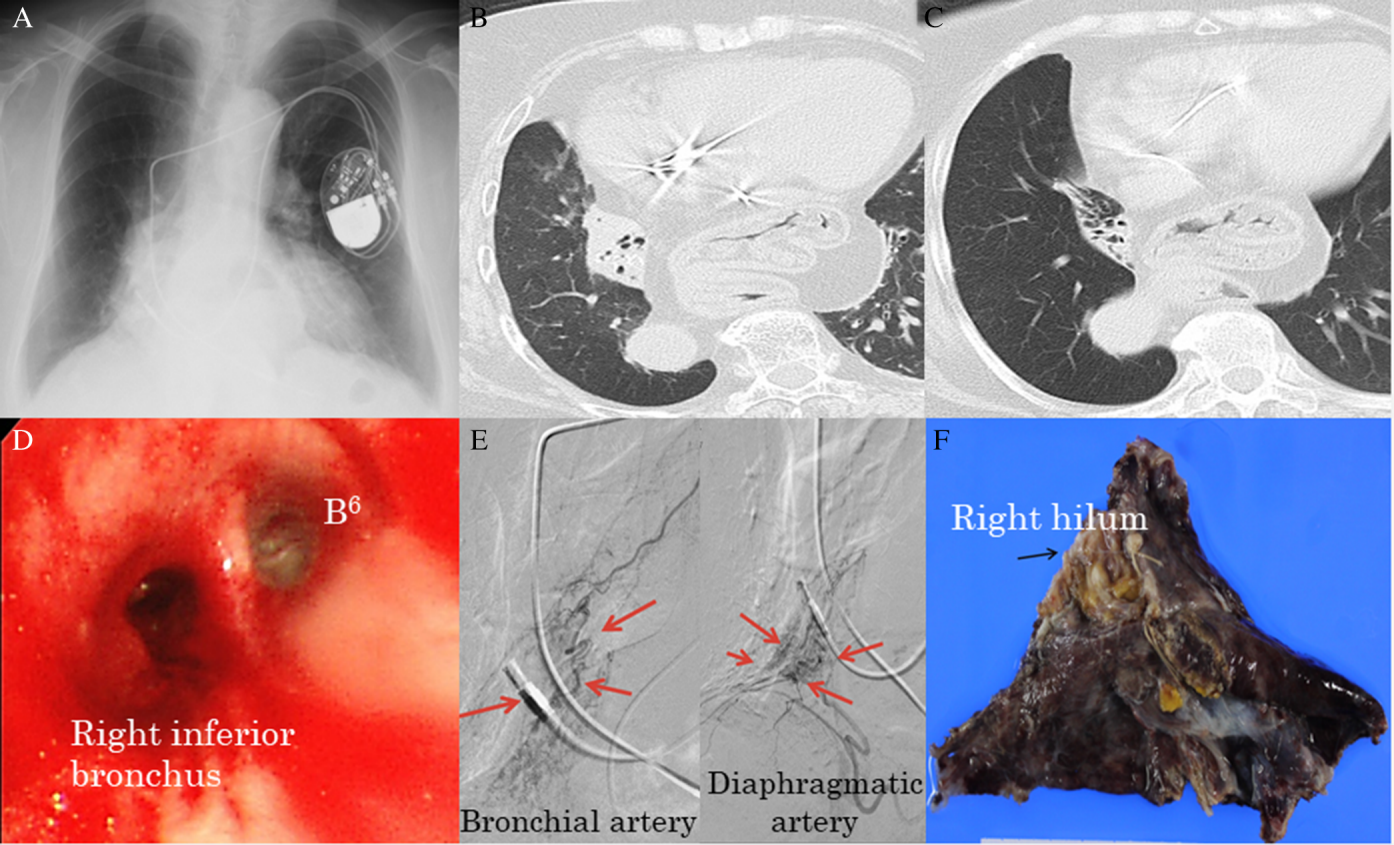
Chest radiograph showed a restiform shadow in the right middle to lower lung field (A). Chest computed tomography (CT) showed a solid nodule in the right lower lobe at the time of admission (B). Chest CT performed five years ago showed atelectasis in the small right lower lobe (C). Bronchoscopy showed the right inferior bronchus completely filled with blood clots (D). The bronchial artery angiography showed the expansion and meandering of the bronchial arteries and the growth of the reticular artery from the diaphragm arteries (E). Macroscopic appearance of the right middle lobe and resected tumour (F).

**Figure 2:**
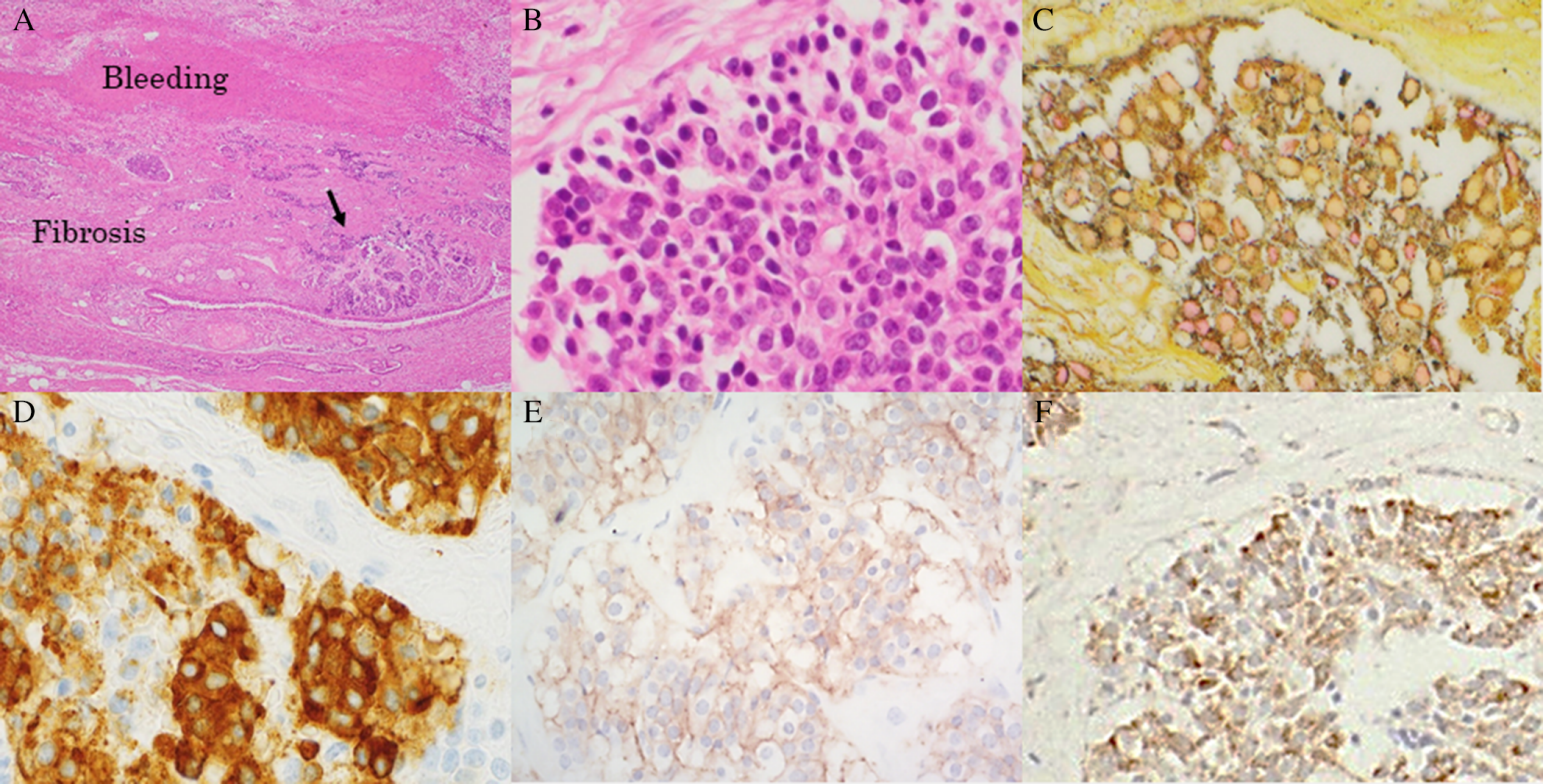
Histological findings of the resected specimen in the right lower lobe. Small hyperplasia and bleeding surrounding or obliterating small bronchi or bronchioles [Hematoxylin and eosin stain (HE) ×4] (A). The tumour was composed of uniform, oval‐shaped cells (HE ×40) (B). Immunohistochemical reactivity for Grimelius (C), chromogranin A (D), CD56 (E), and VEGF (F) (×200).

A 73‐year‐old woman visited our hospital with the complaint of coughing up blood for three days. She had experienced massive haemoptysis the previous year, but no disorder could be identified during her follow‐up examination. The patient had a significant past medical history of hypertension, hyperlipidaemia, and atrioventricular block with an implanted dual chamber pacemaker and a negative smoking history. On examination in the emergency room, her blood pressure was 146/72 mmHg, pulse was 86 beats/min (regular), temperature was 36.6 °C, respiratory rate was 20/min, and blood oxygen saturation level was 95% on room air. Her physical examination was unremarkable, without coarse crackles on the right lung field, and laboratory test results were within the normal limits. Her chest radiograph showed a homogenous, flat opacity along the right heart border in the lower lung field (Fig. 1A). Chest computed tomography (CT) demonstrated only a consolidation due to a collapsed right lower lobe and a diaphragmatic hernia (Fig. 1B). A chest CT performed five years ago showed an expanded bronchus in the small right lower lobe; these findings suggested pulmonary hypoplasia of the right inferior lobe (Fig. 1C). In the dynamic CT angiography, we could not detect the extravasation of blood on arterial and venous phase; however, bronchoscopic examination demonstrated that the right inferior lobar bronchus was filled with blood clots (Fig. 1D). The bronchial artery angiography confirmed the expansion and meandering of the bronchial arteries and the growth of the reticular artery from the diaphragm arteries (Fig. 1E). We performed two arterial embolizations and also plugged an endobronchial Watanabe spigot into her right inferior lobar bronchus but failed to stop the haemoptysis. Therefore, we were forced to perform a thoracoscopic right lower lobectomy. The excised surgical specimen presented a small hypoaerated, atrophied, and occluded right inferior lobe. The bronchus was underdeveloped and irregular throughout its running (Fig. 1F). Microscopic examination demonstrated extensive small epithelial nests surrounding the blood and fibrosis in the bronchial mucosa. These multiple micronodular hyperplastic foci were smaller than 5 mm in diameter (Fig. 2A). These tumorlets were detected around the erosional mucous membrane of bronchus. The nodules were composed of a relatively uniform population of atypical cells with moderate amounts of scant cytoplasm and oval nuclei without mitotic activity and exhibited necrosis beneath the bronchial mucosa (Fig. 2B). There were no histological features to suggest malignancy. Immunohistochemical markers stained positively for Grimelius and chromogranin A, CD56, and vascular endothelial growth factor (Fig. 2C–F). These findings led us to the diagnosis of pulmonary tumorlets on a hypoplastic right lower lobe. The postoperative course was uneventful, and the patient was eventually discharged.

## Discussion

Patients with pulmonary tumorlets are relatively asymptomatic or present with cough and recurrent pulmonary infections. Pulmonary function test results for most patients are either normal or mildly abnormal. The majority are non‐smokers. Tumorlets have been more frequently reported in women aged 60–70 years 1. They are usually incidental findings during histopathological lung examinations or after surgery. Although patients have persistent symptoms or lesions, they have a good prognosis without specific adjuvant therapy 1. In our patient, we observed the progression of a decreasing alveolar air space in the right lower lobe over at least five years. Furthermore, the patient was experiencing a life‐threatening haemoptysis at the time of admission. There have been few reports on the acute progression of lung tumorlets.

In this case, the tumorlets were observed in the hypoplastic right inferior lobe. Pulmonary hypoplasia is a rare congenital malformation. It occurs most often secondary to other foetal abnormalities that interfere with normal development of the lungs, such as diaphragmatic hernia. Pulmonary hypoplasia is a relatively common cause of neonatal death; however, some patients exhibit a benign course, and the condition is often discovered accidentally. Adult patients may be relatively asymptomatic, but others present with repeated pulmonary infections and wheezing 2. There are no reports of pulmonary hypoplasia with haemoptysis. We found a report of neuroendocrine cell hyperplasia complicated with pulmonary hypoplasia 3 but found no cases of lung tumorlets complicated with pulmonary hypoplasia. The relationship between lobar hypoplasia and lung tumorlets is unclear.

Essentially, few neuroendocrine cells are found within the bronchial and bronchiolar epithelium of the normal lung tissue. Tumorlets develop from these neuroendocrine cells, known as Kulchitsky cells. The pulmonary neuroendocrine cells induce the hyperplastic cells of tumorlets as a consequence of chronic lung diseases, such as inflammation or fibrosis, resulting in hypoxia or bleeding 4. Our data suggest that chronic inflammation may have stimulated the production of pro‐fibrotic growth factors from tumorlets in the present case as evidenced by the detection of vascular endothelial growth factor (VEGF) expression in the tumorlets using immunohistochemistry (Fig. 2F). According to the report of Sartelet et al., VEGF R1 and VEGF R2 are expressed in both tumorlets and neuroendocrine cell hyperplasia 5. Our case study suggests that VEGF secretion by tumorlets causes lung tissue inflammation, inducing repeated haemoptysis.

However, the possibility that chronic inflammation due to pulmonary hypoplasia caused tumorlet development cannot be excluded; further studies involving more patients are needed to clarify our case findings. We described a case of lung tumorlets with pulmonary hypoplasia. In spite of the rarity of tumorlet cases, we suggest that lung tumorlets should be included in the differential diagnosis for patients complaining of recurrent haemoptysis.

### Disclosure Statement

Appropriate written informed consent was obtained for publication of this case report and accompanying images.
